# Association of serum brain-derived neurotrophic factor with hepatic enzymes, AST/ALT ratio, and FIB-4 index in middle-aged and older women

**DOI:** 10.1371/journal.pone.0273056

**Published:** 2022-08-23

**Authors:** Takumi Yokokawa, Shohei Sasaki, Kohei Sase, Naomi Yoshii, Jun Yasuda, Tatsuya Hayashi, Satoshi Fujita

**Affiliations:** 1 Laboratory of Sports and Exercise Medicine, Graduate School of Human and Environmental Studies, Kyoto University, Kyoto, Japan; 2 Division of Food Science and Biotechnology, Graduate School of Agriculture, Kyoto University, Kyoto, Japan; 3 Faculty of Sport and Health Science, Ritsumeikan University, Shiga, Japan; 4 Japan Institute of Sports Sciences, Tokyo, Japan; Chiba Daigaku, JAPAN

## Abstract

Substantial evidence suggests an important role of liver function in brain health. Liver function is clinically assessed by measuring the activity of hepatic enzymes in the peripheral blood. Brain-derived neurotrophic factor (BDNF) is an important regulator of brain function. Therefore, we hypothesized that blood BDNF levels are associated with liver function and fibrosis. To test this hypothesis, in this cross-sectional study, we investigated whether serum BDNF concentration is associated with liver enzyme activity, aspartate aminotransferase (AST)/ alanine aminotransferase (ALT) ratio, and fibrosis-4 (FIB-4) index in middle-aged and older women. We found that serum BDNF level showed a significant positive association with ALT and γ-glutamyltranspeptidase (GGT) activity and negative association with FIB-4 index, and a trend of negative association with the AST/ALT ratio after adjustment for age. Additionally, these associations remained statistically significant even after adjustment for body mass index (BMI) and fasting blood glucose level. These results demonstrate associations of serum BDNF levels with liver enzymes and hepatic fibrosis-related indices, which may underlie liver-brain interactions.

## Introduction

There is substantial evidence suggesting an important role of liver function in brain health. Patients with nonalcoholic fatty liver disease (NAFLD) exhibit lower cognitive performance [1]. In NAFLD, fibrosis severity is negatively associated with cognitive function in middle-aged adults [2]. Additionally, cerebral brain atrophy has been observed in middle-aged patients with NAFLD [3]. Biochemical assessment of liver function is performed by measuring the activity of hepatic enzymes, such as alkaline phosphatase (ALP), alanine aminotransferase (ALT), aspartate aminotransferase (AST), γ-glutamyl transpeptidase (GGT), and bilirubin, in the peripheral blood. The enzymatic activities of ALT and AST are negatively associated with cognitive function in adults aged 20–59 years [1]. NAFLD with a higher fibrosis-4 (FIB-4) index is associated with the presence of moderate to severe white matter hyperintensities in cognitively normal individuals [4]. A higher AST/ALT ratio and lower ALT levels were associated with Alzheimer’s disease and poor cognitive performance in older adults [5]. Liver function and hepatic enzymes may therefore play an important role in brain function; however, these physiological mechanisms remain unclear.

Brain-derived neurotrophic factor (BDNF) is a crucial regulator of neuronal development and function. Adult BDNF-knockout mice demonstrated impairment of hippocampal-dependent learning [6]. A clinical study reported that plasma BDNF levels were associated with cognitive impairment in non-demented middle-aged and older women [7]. Furthermore, BDNF levels in the blood, which decrease with age [8, 9], are associated with age-related hippocampal atrophy [9]. Pathologically, serum BDNF levels decrease in patients with Alzheimer’s disease [10], schizophrenia [11], and depression [12]. Systematic reviews and meta-analyses further suggest that circulating BDNF is associated with these conditions [13–15]. Accordingly, appropriate levels of circulating BDNF are important for human brain health and function.

Blood BDNF concentration decreases with aging, type 2 diabetes (T2D), and obesity [8, 16], which are all associated with cognitive impairment [17–20]. Aging is suggested to be a risk factor for liver fibrosis progression [21], as an enhanced liver fibrotic response was observed in older mice [22]. Of note, a recent study reported that individuals who are intermediate or high risk for advanced fibrosis demonstrated lower BDNF levels than those at low risk [23]. Hence, it is plausible that liver dysfunction and fibrosis are associated with blood BDNF levels in older adults, which may be partly involved in age-related brain dysfunction.

We hypothesized that blood BDNF levels are associated with liver function and fibrosis. To test this hypothesis, we investigated the correlation of serum BDNF concentration with the activity of hepatic enzymes and hepatic fibrosis-related indices in healthy middle-aged and older females.

## Results

### Characteristics of the participants

Sixty-five participants were included in this study, and their characteristics are shown in Table 1. The median age was 61 years; 45 participants (69.2%) were middle-aged women (45–64 years), while 20 (30.8%) were older women (65 years and older). Seven participants (10.8%) were overweight (BMI ≥ 25 kg/m^2^), while none of the participants were obese (BMI ≥ 30 kg/m^2^). One participant (1.5%) showed an increased fasting glucose level based on the cut-off value for the Japanese population (fasting blood glucose level ≥ 113 mg/dL) [24]. Based on the cut-off values, one participant (1.5%) showed high levels of ALP (≥ 350 IU/L) and total bilirubin (≥ 1.2 mg/dL), two (3.1%) demonstrated high levels of ALT (≥ 30 IU/L), and six (9,2%) had elevated GGT activity (≥ 40 IU/L). In contrast, all participants had normal levels of AST (< 30 IU/L). A high FIB-4 index was observed in 23 participants (35.4%). This increased rate of high FIB-4 indices is because this index is calculated based on age [25]. The median serum BDNF concentration was 17.3 ng/mL, which was comparable to a previous study [10].

**Table 1:** Characteristics of the participants.

	median (IQR)
Age, years	61 (54–65)
BMI, kg/m^2^	21.6 (20.1–23.7)
Fasting glucose, mg/dL	88 (83–93)
ALT, IU/L	15 (13–19)
AST, IU/L	21 (18–24)
ALP, IU/L	220 (195–257)
Total bilirubin, mg/dL	0.6 (0.5–0.8)
GGT, IU/L	18 (14–23)
Platelet numbers, ×10^9^/L	241 (209–288)
AST/ALT ratio	1.33 (1.13–1.50)
FIB-4 index	1.32 (1.04–1.59)
Serum BDNF, ng/ml	17.3 (13.0–22.4)

Abbreviations: BMI, body mass index; ALT, alanine aminotransferase; AST, aspartate aminotransferase; ALP, alkaline phosphatase; GGT, γ-glutamyl transpeptidase; FIB-4, fibrosis-4; BDNF, brain-derived neurotrophic factor; IQR, interquartile range

### Association of serum BDNF level with liver enzyme activity in the blood

After adjusting for age, higher serum BDNF levels were significantly associated with higher ALT (β = 6.120, 95% confidence interval (CI) 0.977 to 11.263, p = 0.020) and GGT activities (β = 3.147, 95% CI 0.180 to 6.114, p = 0.038) (Table 2, Model 1). Despite adjusting for BMI and fasting glucose, associations with ALT (β = 6.920, 95% CI 1.518 to 12.322, p = 0.012) and GGT activity (β = 4.214, 95% CI 0.895 to 7.532, p = 0.014) remained statistically significant (Model 2). In contrast, we did not find any significant relationship between serum BDNF levels, ALP and AST activity, and total bilirubin levels after adjustments (Model 1 and 2).

**Table 2:** Results of association of serum BDNF level with liver enzymes.

	Model	β	95% CI	p value
AST	Model 1	0.331	-0.111 to 0.772	0.140
	Model 2	0.328	-0.126 to 0.783	0.153
ALT	Model 1	6.120	0.977 to 11.263	0.020
	Model 2	6.920	1.518 to 12.322	0.012
ALP	Model 1	0.011	-0.019 to 0.042	0.463
	Model 2	0.015	-0.018 to 0.048	0.369
Total bilirubin	Model 1	0.321	-5.322 to 5.963	0.910
	Model 2	0.335	-5.457 to 6.128	0.908
GGT	Model 1	3.147	0.180 to 6.114	0.038
	Model 2	4.214	0.895 to 7.532	0.014

Model 1 was adjusted for age. Model 2 was adjusted for age, BMI, and fasting glucose level.

Abbreviations: β, regression coefficients; AST, aspartate aminotransferase; ALT, alanine aminotransferase; ALP, alkaline phosphatase; GGT, γ-glutamyl transpeptidase; CI, confidence interval

### Association of serum BDNF level with AST/ALT ratio and FIB-4 index

After adjusting for age, serum BDNF levels were significantly associated with the FIB-4 index (β = -6.439, 95% CI -11.067 to -1.810, p = 0.007) and showed a trend of association with the AST/ALT ratio (β = -4.554, 95% CI -9.990 to 0.882, p = 0.099) (Table 3, Model 1). Similarly, serum BDNF level was significantly related to a lower AST/ALT ratio (β = -6.139, 95% CI -12.220 to -0.057, p = 0.048) and FIB-4 index (β = -6.726, 95% CI -11.473 to -1.980, p = 0.006), even after further adjustment for BMI and fasting glucose (Model 2).

**Table 3:** Results of association of serum BDNF level with AST/ALT ratio and FIB-4 index.

	Model	β	95% CI	p value
AST/ALT ratio	Model 1	-4.554	-9.990 to 0.882	0.099
	Model 2	-6.139	-12.220 to -0.057	0.048
FIB-4 index	Model 1	-6.439	-11.067 to -1.810	0.007
	Model 2	-6.726	-11.473 to -1.980	0.006

Model 1 was adjusted for age. Model 2 was adjusted for age, BMI, and fasting glucose level.

Abbreviations: β, regression coefficients; AST, alkaline phosphatase; ALT, aspartate aminotransferase; FIB-4, fibrosis-4; CI, confidence interval

## Discussion

In this cross-sectional study, we investigated the association of serum BDNF concentration with liver enzyme activity, AST/ALT ratio, and FIB-4 index in 65 middle-aged and older women. We found that serum BDNF levels were positively associated with ALT and GGT activity, and negatively associated with AST/ALT ratio and FIB-4 index after adjusting for age, BMI, and fasting blood glucose level. This is the first study showing the associations of serum BDNF levels with liver enzymes and hepatic fibrosis-related indices, which may underlie liver-brain interactions.

The hepatic state is closely connected to brain health, such as cerebral brain volume [3], cognitive function [1, 2], neurodegenerative disorders [5], and depression [26]. A previous study reported that a higher AST/ALT ratio and lower ALT levels were associated with Alzheimer’s disease and poor cognitive performance in older adults [5]. Another study has shown that NAFLD with higher FIB-4 levels is associated with the presence of moderate to severe white matter hyperintensities in cognitively normal individuals [4]. Nevertheless, the mechanism underlying the liver-brain interaction remains uncertain. Interestingly, a recent study reported that patients with intermediate or high risk for advanced fibrosis showed lower BDNF levels than those with lower risk [23]. In contrast, no study has investigated whether this relationship can be observed in older adults without NAFLD and whether serum BDNF levels are associated with hepatic enzyme activities and fibrosis-related indices. Our results demonstrate that serum BDNF levels are positively associated with AST and GGT activity and negatively associated with AST/ALT ratio and FIB index in healthy middle-aged and older women. Lower circulating BDNF levels are associated with cognitive impairment [7] and age-related hippocampal atrophy [9]. In pathological states, serum BDNF levels decrease, such as in patients with Alzheimer’s disease [10, 15], schizophrenia [11, 14], and depression [12, 13]. Thus, BDNF may be responsible for the association of altered hepatic enzymes and hepatic fibrosis-related indices with brain function and health. However, in this study, we did not evaluate cognitive function; therefore, further comprehensive studies are required to measure and investigate the relationships of BDNF levels, liver enzyme activities, fibrosis-related indices, and cognitive function.

Hepatic function and fibrosis are closely associated with obesity and T2D [27, 28], both of which cause cognitive impairment [29, 30]. In this context, blood BDNF levels are decreased in T2D and obese patients [16]. As a physiological mechanism, enhanced levels of glucose, but not insulin, inhibit the release of BDNF from the brain into circulation [16]. Thus, we investigated whether the relationship between serum BDNF levels, hepatic enzymes, and fibrosis-related indices is an epiphenomenon associated with obesity and blood glucose levels. In this study, these significant associations were observed even after adjusting for age, BMI, and fasting glucose level, suggesting that serum BDNF levels are independently associated with ALT and GGT activity, AST/ALT ratio, and FIB-4 index in the healthy population.

The physiological mechanism underlying the association of hepatic function and fibrosis with serum BDNF levels remains unclear. A previous study reported that female patients with NAFLD showed reduced brain activity during verbal fluency tasks [31]. Neural activity regulates the expression of human BDNF [32], which can be transported across the blood-brain barrier [33]. Hence, neural activity may mediate between liver function and serum BDNF levels. Alternatively, a recent study suggested that a liver-derived glycosylphosphatidylinositol-specific phospholipase D1 (Gpld1) induces BDNF expression and neurogenesis in the hippocampus, which subsequently improves learning impairment in aged mice [34]. Therefore, Gpld1 or other unidentified hepatokines may mediate the association of liver state with BDNF and brain function. Moreover, although BDNF has been demonstrated to cross the blood brain barrier [33], serum BDNF levels may not completely reflect its expression in brain; thus, it is required to investigate direct association of liver state with BDNF expression in the brain. Taken together, further investigation is required to verify the mechanism underlying the association of hepatic function and fibrosis with BDNF and brain function.

In this study, we have preferentially investigated female because 1) serum BDNF levels were associated with learning in female [7] and 2) female patients with NAFLD showed reduced brain activity during verbal fluency tasks [31]. On the other hand, previous studies indicate association between liver indices and cognitive function after adjustment for gender [1]. Therefore, serum BDNF levels may be associated with liver markers in male, suggesting that further studies that recruit both genders are required.

In addition to the aforementioned limitations, our study had several others. First, because this study had a cross-sectional design, it did not permit the determination of a temporal sequence, and causal relationships could not be inferred. Interestingly, BDNF protein is observed in the human liver, suggesting a brain-liver axis [35]. In obese mice, administration of a BDNF mimetic suppresses lipid accumulation in the liver and improves insulin resistance [36]. In contrast, another study demonstrated that BDNF treatment does not change the ALT/AST ratio in diabetic mice [37]. It should be noted that the expression of its receptor TrkB is limited in the human liver [38]. Hence, further studies are required to verify the causal relationships. Second, because of the small sample size, we could not firmly adjust for confounding factors, such as blood pressure, endothelial function, and cardiovascular function. Third, the study population was limited to Japanese women aged between 51 and 73 years from one geographic area; thus, the external validity for other ethnicities and populations is uncertain. Therefore, our results should be cautiously interpreted and warrant further replication in other larger prospective studies. Finally, the ELISA kit we used in this study recognizes precursor and mature BDNF, both of which exist in human blood [39, 40]; thus, our present study fails to discriminate mature BDNF from its precursor. This discrimination in further studies may provide novel molecular insights into the relationship between BDNF and hepatic function.

In conclusion, our results demonstrate associations of serum BDNF levels with liver enzymes and hepatic fibrosis-related indices, which may underlie liver-brain interactions. Further comprehensive studies are required to verify the detailed mechanisms underlying this association.

## Methods

### Participants and ethical approval

A total of 65 healthy females aged 51–73 years were enrolled in this cross-sectional study. The inclusion criteria were (1) women aged 50–79 years, (2) BMI 25–30, and (3) exercise habit less than once a week and no experience in resistance training. The exclusion criteria were (1) those with serious or progressive pre-existing illness or disease, including psychiatric disorders and alcoholism, and (2) those with food allergies. The study protocol was approved by the Ethics Committee for Human Experiments at Ritsumeikan University (BKC-IRB-2014-039) and was conducted in accordance with the Declaration of Helsinki. After recruiting participants through newspaper advertisements, an information session was held to explain the study in writing and orally, and then the participants signed a consent form.

### Anthropometric measurements

Weight, height, and body fat mass were measured, and body mass index (BMI; body weight [height]^2^ kg/m^2^) was calculated.

### Blood analysis

Blood samples were collected from a vein in the cubital fossa at rest in a fasting state and then centrifuged at 3000 rpm at 4°C for 10 min with a KUBOTA5500 and ST-720M rotor (Kubota, Tokyo, Japan). The separated serum samples were frozen and stored at -80°C until analysis. Serum BDNF concentration was determined using enzyme-linked immunosorbent assay (ELISA) kits (Abcam, Cambridge, UK, ab212166), following the manufacturer’s instructions. The absorbance was measured using a microplate reader (xMark microplate spectrophotometer, Bio-Rad Laboratories, Hercules, CA, USA). The enzymatic activities of AST, ALT, ALP, GGT, total bilirubin, platelet numbers, and glucose concentration were determined by KINKIYOKEN, Inc., Shiga, Japan. The FIB-4 values were calculated using the formula: age (years) × AST (IU/L) / (platelets [10^9^/L] × [ALT [IU/L])^1/2^).

### Statistical analysis

All statistical analyses were performed using the R software. Linear regression models were constructed to estimate beta coefficients for the association between serum BDNF levels and hepatic enzyme activities and liver fibrosis. We used two adjustment models. The basic model (model 1) was adjusted only for age. Model 2 was adjusted for age, BMI, and fasting blood glucose levels. To normalize the residuals, age, fasting glucose level, ALT, total bilirubin, GGT, and FIB-4 index were logarithmically transformed. In all models, collinearity was assessed using the variance inflation factor with a cut-off of 10. The statistical significance level was set at p < 0.05.

## Supporting information

S1 Table Dataset. (PDF)

S1 Text (TXT)
