# Supplementary material for: Association of serum brain-derived neurotrophic factor with hepatic enzymes, AST/ALT ratio, and FIB-4 index in middle-aged and older women
Source: PLoS One. 2022 Aug 23;17(8):e0273056. doi: 10.1371/journal.pone.0273056 (PMC9398011; doi:10.1371/journal.pone.0273056)
Supplement: S1 Table — (PDF) [file pone.0273056.s001.pdf]

No.	Serum BDNF, ng/ml	Age, years	height, cm	weight, kg	Platelet, $\times 10^9$/L	GGT, IU/L	ALP, IU/L	ALT, IU/L	AST, IU/L	Total bilirubin, mg/dL	Fasting glucose, mg/dL
#1	23.95844	61	151.3	51.4	213	17	333	18	22	0.4	94
#2	25.40452	54	159.6	64.75	351	90	310	32	22	0.6	90
#3	9.78644	65	156.8	49.8	285	13	250	16	21	0.7	82
#4	7.58604	65	155.8	51.8	169	21	170	19	20	0.8	89
#5	7.55224	62	163.2	70.7	123	19	123	24	24	0.7	92
#6	16.22276	57	153.7	46.55	241	19	192	18	24	0.6	81
#7	10.13708	69	149	52.5	263	18	257	16	24	0.8	96
#8	21.61164	54	157.6	50.7	173	23	208	20	26	1.3	82
#9	15.7122	54	161.3	45.85	222	16	172	15	21	0.8	85
#10	11.20716	72	146.7	52.6	174	14	206	16	24	0.7	88
#11	24.348	53	157.8	55.3	225	12	177	12	20	0.8	84
#12	16.37496	63	158.7	50.55	207	14	139	18	23	0.6	82
#13	26.0994	61	157.5	59.35	310	42	271	35	24	0.5	85
#14	14.24584	64	154.1	52.75	173	15	231	13	24	0.6	86
#15	13.56604	62	150.9	46.55	219	18	253	14	20	0.5	82
#16	9.34284	65	157.3	44.6	401	14	292	13	24	0.3	94
#17	24.90672	61	156.4	48.4	315	46	212	28	30	0.6	87
#18	21.56452	64	150.6	48.85	365	11	251	12	20	0.5	81
#19	25.8304	62	153.6	49.25	256	14	182	13	16	0.8	77
#20	15.91888	52	155.4	61.9	201	181	232	23	25	0.5	94
#21	22.76676	53	155.3	48.5	320	13	233	11	17	0.6	91
#22	15.91084	66	148.6	50.95	264	16	175	12	17	0.6	88
#23	21.25628	62	159.5	48.05	221	32	199	16	29	1	85
#24	4.73928	60	160.7	50.8	215	16	214	13	19	0.5	90
#25	26.81644	67	157.7	56.05	294	34	266	20	20	1.2	83
#26	24.22336	66	163.2	54.8	256	21	202	22	26	0.5	96
#27	5.72892	51	160.7	56.35	191	14	198	13	18	1.2	89
#28	13.49552	61	161	62.95	252	18	207	16	19	0.4	92
#29	9.00268	73	148.1	63.05	281	36	277	15	20	0.4	85
#30	31.13124	58	155.5	51.35	314	29	220	28	23	0.6	84
#31	22.39172	66	159.8	56.35	306	26	256	15	21	0.6	95
#32	29.43288	71	146.4	49.3	276	16	214	14	20	0.6	89
#33	21.76224	62	155	54.6	186	42	211	22	24	0.7	82
#34	13.14548	61	152.4	53.45	215	20	261	15	17	0.8	88
#35	11.01968	54	151.9	49.3	214	13	232	14	19	0.5	87
#36	12.54796	66	153.8	63.95	280	21	304	21	17	0.7	88
#37	18.07184	54	162	50.65	197	10	202	14	24	0.6	80
#38	16.96408	65	153.4	50.65	398	14	240	16	21	0.7	89
#39	22.64644	58	157.1	60.05	301	28	195	17	17	0.8	91
#40	9.54244	53	164.1	50.45	209	10	213	13	16	1	79
#41	17.28432	62	150.8	44.8	244	21	181	16	25	0.4	93
#42	20.04544	63	159.3	47.15	201	15	174	19	24	0.6	72
#43	22.43212	59	155.7	47.75	198	14	295	14	22	0.6	111
#44	22.33136	51	157.8	53.85	328	12	160	12	17	0.7	88
#45	21.9946	66	151.2	58.05	349	35	178	28	22	0.4	99
#46	18.4458	58	158.2	48.7	258	15	163	11	18	0.6	96
#47	11.11876	54	165.8	68.65	219	18	222	16	20	0.7	88
#48	23.97976	57	153.3	48.1	318	23	200	16	18	0.6	100
#49	16.81668	65	150.6	60.1	150	42	327	23	28	0.9	81
#50	10.31588	59	160.1	54.75	222	16	257	18	22	0.6	94
#51	13.11748	70	153.9	59	231	11	276	12	20	0.7	76
#52	17.77124	53	160	56.35	252	18	257	10	14	0.9	103
#53	18.32224	67	158.5	48.75	247	18	204	27	27	0.8	92
#54	7.58032	65	157.6	58.35	259	20	173	15	16	0.5	93
#55	13.02916	60	160.3	50.1	178	14	239	14	26	0.4	82
#56	10.22992	60	162	62.2	216	10	284	11	18	0.5	78
#57	21.44964	61	156.6	61	225	25	396	28	23	0.6	90
#58	13.93756	67	159.7	54.35	228	15	221	11	17	0.6	114
#59	19.27764	53	160.6	57.3	288	15	192	12	17	0.5	90
#60	21.5624	54	158.1	61.4	282	16	305	15	21	0.8	88
#61	24.48048	67	149.4	49.9	333	10	241	10	17	0.4	83
#62	23.55284	56	159	61.7	131	23	293	22	27	0.7	103
#63	15.89224	53	151.9	47.3	211	21	287	14	17	0.9	102
#64	16.1422	62	158.3	48.15	173	10	148	13	21	0.7	91
#65	23.13964	51	160	53.85	309	23	204	13	19	0.7	84
